# Groundwater quality assessment for irrigation purposes based on irrigation water quality index and its zoning with GIS in the villages of Chabahar, Sistan and Baluchistan, Iran

**DOI:** 10.1016/j.dib.2018.05.061

**Published:** 2018-05-18

**Authors:** Abbas Abbasnia, Majid Radfard, Amir Hossein Mahvi, Ramin Nabizadeh, Mahmood Yousefi, Hamed Soleimani, Mahmood Alimohammadi

**Affiliations:** a Department of Environmental Health Engineering, School of Public Health, Tehran University of Medical Sciences, Tehran, Iran; b Center for Water Quality Research (CWQI), Institute for Environmental Research (IER), Tehran University of Medical Sciences, Tehran, Iran

**Keywords:** Groundwater quality, IWQI, GIS, Chabahr, Iran

## Abstract

The present study was conducted to evaluate the groundwater quality and its suitability for irrigation purpose through GIS in villages of Chabahr city, Sistan and Baluchistan province in Iran. This cross-sectional study was carried out from 2010 to 2011 the 1-year-monitoring period. The water samples were collected from 40 open dug wells in order to investigate the water quality. Chemical parameters including EC, SAR, Na^+^, Cl^−^, pH, TDS, ${HCO}_{3}^{-}$ and IWQI were analyzed. In order to calculate the irrigation water quality index subsequent five water quality parameters (EC, SAR, Na^+^, Cl^−^, and ${HCO}_{3}^{-}$) were utilized. Among the total of 40 samples were analyzed for IWQI, 40% of the samples classified as excellent water, 60% of the samples in good water category.

**Specifications Table**

**Table:** 

Subject area	Chemistry
More specific subject area	Describe narrower subject area
Type of data	Table, Graph, Figure
How data was acquired	Using polythene bottles (1L) samples were collected and then transported to the central laboratory of the water and wastewater company. Groundwater samples were collected and transported to the laboratory on the same day and kept at 4 °C. All water samples were analyzed according to the Standard Methods for Examination of Water and Wastewater Temporary. Also permanent magnesium, calcium, and chloride were measured using titration method. pH meter (model wtw, Esimetrwb) and turbidity meter (model Hach 50161/co 150 model P2100Hach, USA) are used to determine the concentration of hydrogen ion (pH) and electrical conductivity, respectively
Data format	Raw, analyzed
Experimental factors	*The mentioned parameters above, in abstract section, were analyzed according to the standards for water and wastewater treatment handbook.*
Experimental features	*The levels of* physical and chemical parameters were determined.
Data source location	Chabahar, Sistan and Balouchestan province,Iran
Data accessibility	*Data are included in this article*

**Values of the data**

• IWQI concept was introduced to determine the suitability of groundwater for irrigation purposes primarily developed by Meireles et al. [5]. Accordingly, the five parameters including EC, SAR, Na^+^, Cl^−^, and ${HCO}_{3}^{-}$ which dominantly influence the water quality for irrigational use were considered for computing IWQI.
• The result of calculated indices shows that water in subjected area is suitable for agricultural uses.
• The results of groundwater quality for irrigational uses were zoned in spatial distribution maps using GIS.

## 1 Data

**Fig. 1:**
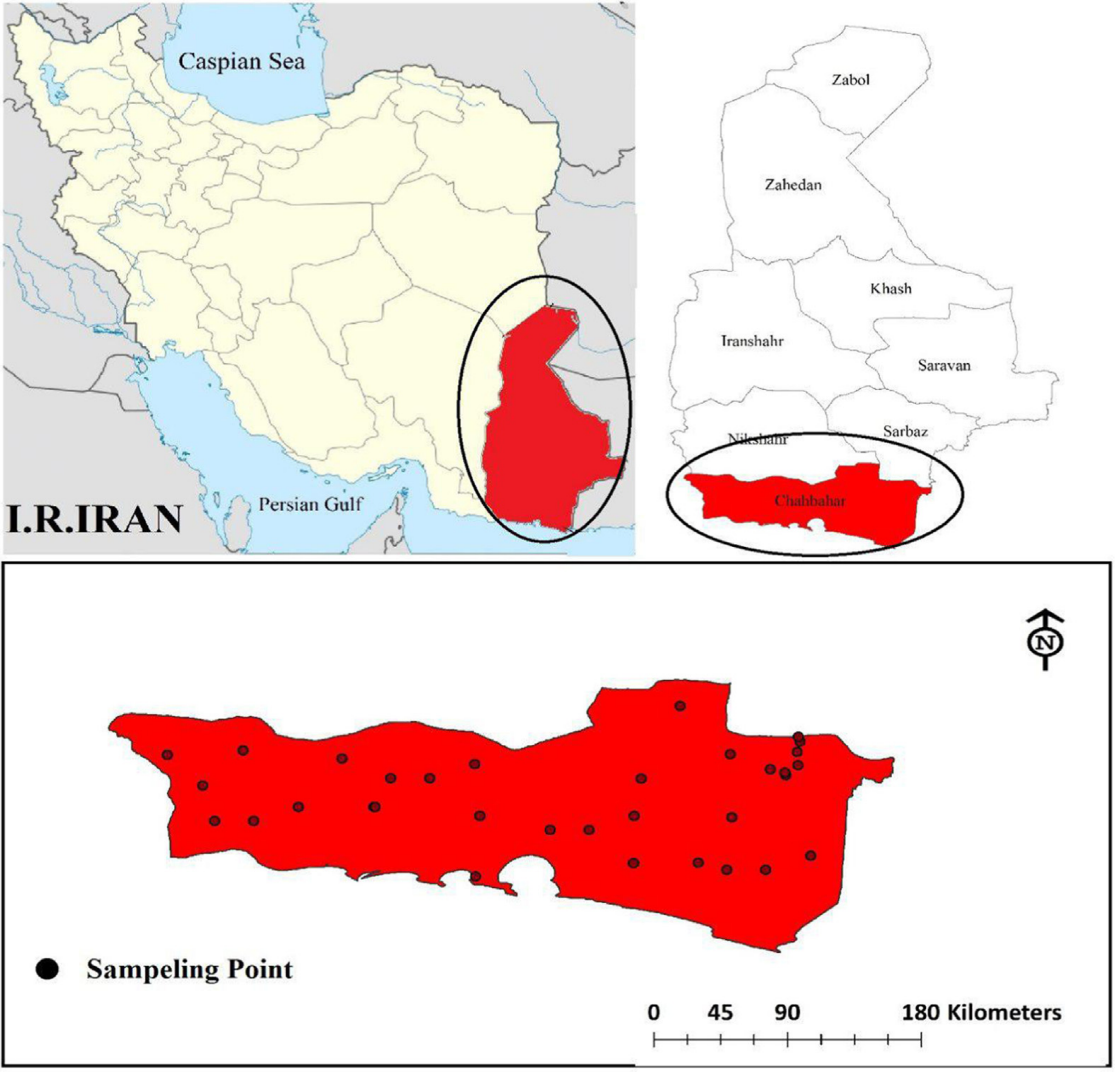
Location of water sampling sites in Chabahar city.

In accord with standard methods, water and wastewater quality parameters were calculated in present study which are including chloride ion, Electrical Conductivity (EC), Total Dissolved Solids (TDS), bicarbonate ions, calcium, and magnesium [1], [2], [3]. The sampling locations are illustrated in Fig. 1. Also the calculated physical and chemical properties of drinking water are presented in Table 1. Qi, X and Wi of individual parameters along with Irrigation Water Quality Index (IWQI) were shown in Table 2. Limiting values for each parameter for quality measurements (*q_i_*) and relative weight (*w_i_*) in IWQI was shown in Table 3, Table 4, respectively.

**Table 1:** Statistics of physico-chemical characteristics and hydro-geochemistry of water quality.

**Number** Well	**Ca**^**2+**^mg/L	**Mg**^**2+**^mg/L	**Na**^**+**^mg/L	**Cl**^−^mg/L	${HCO}_{3}^{-}$mg/L	**TDS** mg/L	**EC (μmhos/cm)**	**SAR** meq/L
W1	78.4	15.84	5.09	6.25	3.28	680.32	1063	3.15
W2	60	24	3.48	4.9	2.32	540.8	845	2.21
W3	64	34.08	3.91	4.82	3.32	588.8	920	2.26
W4	100	36	16.52	10.23	5	1478.4	2310	8.29
W5	64.8	29.28	16.96	10.62	4.08	1484.8	2320	10.1
W6	72	24.96	4.35	5.55	2.12	593.28	927	2.59
W7	49.6	20.64	4.57	3.21	3.56	567.04	886	3.16
W8	80	34.08	16.09	8.06	4.32	1427.2	2230	8.73
W9	64.8	24.96	3.91	2.2	4.16	545.92	853	2.41
W10	136	28.8	5	5.18	5.12	917.76	1434	2.34
W11	144	48	16.96	14.23	6.4	1824	2850	7.19
W12	121.6	30.72	23.35	11.01	6.32	2080	3250	11.26
W13	153.6	37.44	18.52	10.56	4.84	1772.8	2770	7.99
W14	44.8	11.52	5.43	3.15	3.68	531.84	831	4.31
W15	124.8	37.92	18.91	11.21	5.04	1772.8	2770	8.75
W16	100	42.72	16.52	10.23	4.56	1625.6	2540	8.01
W17	64.8	21.12	3.7	2.42	3.96	534.4	835	2.34
W18	80.8	23.52	5.22	4.31	4.84	766.08	1197	3.02
W19	158.4	74.88	19.13	13.61	6.72	2009.6	3140	7.22
W20	153.6	42.72	13.91	9.77	5.12	1676.8	2620	5.88
W21	41.6	12.96	5.04	2.65	3.44	502.4	785	4.02
W22	113.6	36.96	14.13	7.44	5.04	1376	2150	6.77
W23	145.6	52.32	11.3	18.68	2.8	1433.6	2240	4.7
W24	136	71.04	4.22	6.82	4.48	1004.8	1570	1.68
W25	144	28.8	5.65	4.99	2.6	956.8	1495	2.59
W26	44	38.8	10.43	10.99	3.56	995.84	1556	6.36
W27	240	52.8	11.3	13.38	0.36	1702.4	2660	3.96
W28	322.4	92.16	4.09	5.55	4.44	1734.4	2710	1.19
W29	409.6	44.16	5.22	5.72	5.44	1721.6	2690	1.5
W30	359.98	29.76	10.17	7.8	3.92	1262.72	1973	3.18
W31	428	39.36	4.39	5.8	5.36	1708.8	2670	1.25
W32	56	16.8	13.35	10.2	3.44	1059.84	1656	9.24
W33	76.8	22.08	9.78	6.59	2.88	947.84	1481	5.82
W34	189.6	25.92	8.26	6.7	2.8	1213.44	1896	3.43
W35	200.8	48.48	14.35	12.17	36.19	1715.2	2680	5.42
W36	291.2	61.92	16.52	21.15	3.88	2208	3450	5.27
W37	316	67.2	19.13	20.48	3.12	2400	3750	5.86
W38	132.8	34.08	10.78	7.07	4.72	1225.6	1915	4.96
W39	179.2	16.32	11.87	10.14	2.72	1491.2	2330	5.23
W40	164	26.4	9.13	7.38	3.8	1228.8	1920	4.01
Mean	147.68	36.54	10.52	8.58	4.84	1282.69	2004.2	4.94
Min	41.6	11.52	3.48	2.2	0.36	502.4	785	1.19
Max	428	92.16	23.35	21.15	36.19	2400	3750	11.26
SD	101.81	17.88	5.77	4.62	5.24	529.34	827.1	2.66

**Table 2:** Qi X Wi of individual parameters and Irrigation Water Quality Index (IWQI).

**Number** **well**	*W_i_*×*q_i_* of EC	*W_i_*×*q_i_* of Na^+^	*W_i_*×*q_i_* of Cl^−^	*W_i_*×*q_i_* of HCO_3_^−^	*W_i_*×*q_i_* of SAR	IWQI
W1	15.29	13.08	12.75	12.38	15.78	69.28
W2	17.13	16.36	14.99	14.96	17.78	81.22
W3	16.50	15.48	15.13	12.27	17.51	76.89
W4	10.67	7.14	6.79	11.70	10.08	46.38
W5	10.64	7.14	6.79	10.22	9.08	43.87
W6	16.44	14.59	13.91	15.50	15.74	76.18
W7	16.79	14.14	10.29	11.62	15.76	68.60
W8	10.86	7.14	10.92	9.57	9.84	48.33
W9	17.07	15.48	14.45	10.01	16.70	73.71
W10	12.16	13.26	14.53	11.60	17.08	68.63
W11	9.34	7.14	6.79	10.54	10.68	44.49
W12	7.39	7.14	6.79	10.61	8.44	40.36
W13	9.53	7.14	6.79	11.84	10.24	45.54
W14	17.25	12.38	10.54	11.30	13.59	65.06
W15	9.53	7.14	6.79	11.67	9.82	44.96
W16	10.10	7.14	6.79	12.07	10.23	46.33
W17	17.22	15.91	13.55	10.54	17.08	74.30
W18	14.16	12.81	15.97	11.84	16.03	70.81
W19	7.39	7.14	6.79	10.27	10.67	42.26
W20	9.90	7.14	9.76	11.60	10.62	49.03
W21	17.64	13.18	12.60	11.94	14.14	69.50
W22	11.06	7.14	11.34	11.67	10.92	52.13
W23	10.84	7.14	6.79	13.67	12.85	51.29
W24	12.49	17.34	11.80	9.14	6.62	57.39
W25	11.65	11.93	14.84	14.21	15.74	68.37
W26	12.52	7.14	6.79	11.62	11.14	49.22
W27	9.80	7.14	6.79	7.07	14.25	45.06
W28	9.68	15.12	13.91	9.25	6.62	54.58
W29	9.73	12.81	13.63	11.34	6.62	54.12
W30	11.50	7.14	11.10	10.65	15.72	56.11
W31	9.78	17.34	13.50	11.40	6.62	58.64
W32	12.28	7.14	6.79	11.94	9.55	47.70
W33	11.77	7.14	12.18	13.45	10.74	55.28
W34	11.69	10.45	12.00	13.67	15.25	63.05
W35	9.76	7.14	6.79	7.07	11.49	42.25
W36	7.39	7.14	6.79	10.76	11.77	43.85
W37	7.39	7.14	6.79	12.81	10.66	44.78
W38	11.64	7.14	11.59	11.94	12.36	54.67
W39	10.62	7.14	6.79	13.88	11.85	50.28
W40	11.63	7.14	11.38	10.98	14.16	55.28

**Table 3:** Parameter limiting values for quality measurement (*Q_i_*) calculation [5].

*q*_i_	E.C (μs/m)	SAR ((mmol L^−1^)^0.5^)	Na (meq/L)	Cl (meq/L)	HCO_3_ (meq/L)
85–100	[200,750)	[2,3)	[2,3)	[1,4)	[1,1.5)
60–85	[750,1500)	[3,6)	[3,6)	[4,7)	[1.5,4.5)
35–60	[1500,3000)	[6,12)	[6,9)	[7,10)	[4.5,8.5)
0–35	EC<200 or	SAR<2 or	Na<2 or	Cl<1 or	HCO_3_<1 or
	EC≥3000	SAR≥12	Na≥9	Cl≥10	HCO_3_≥8.5

**Table 4:** weights for the IWQI parameters.

Parameters	*w_i_*
[EC]	0.211
[Na]	0.204
[HCO_3_]	0.202
[CL]	0.194
[SAR]	0.189
Total	1

Division in classes was done based on the water quality index proposed by Bernardo [14] and Holanda and Amorim (1997) in which classes were defined considering the risk of salinity problems, soil water infiltration reduction, as well as toxicity to plants [4]. Also restriction to irrigational water use classes were characterized based on Meireles et al. (Table 5) [5].

**Table 5:** Classifications and characteristics of general IWQI [5].

IWQIM	Exploitation restrictions	Recommendation	
		Soil	Plant
[85,100]	No restriction (NR)	Water can be used for almost all types of soil. Soil is exposed to lower risks of salinity/sodicity problems	No toxicity risk for most plants
[70,85]	Low restriction (LR)	Irrigated soils with a light texture or moderate permeability can be adapted to this range. To avoid soil sodicity in heavy textures, soil leaching is recommended.	Elevated risks for salt sensitive plants
[55,70]	Moderate restriction (MR)	The water in this range would be better used for soils with moderate to high permeability values. Moderate leaching of salts is highly recommended to avoid soil degradation.	Plants with moderate tolerance to salts may be grow
[40,55]	High restriction (HR)	This range of water can be used in soils with high permeability without compact layers. High frequency irrigation schedule	Suitable for irrigation of plants with moderate to high tolerance to salts with special salinity control practices, except water with low Na, Cl and HCO_3_ values
[0,40]	Severe restriction (SR)	Using this range of water for irrigation under normal conditions should be avoided.	Only plants with high salt tolerance, except for waters with extremely low values of Na, Cl and HCO_3_.

## 2 Experimental design, materials and methods

### 2.1 Study area description

**Fig. 2:**
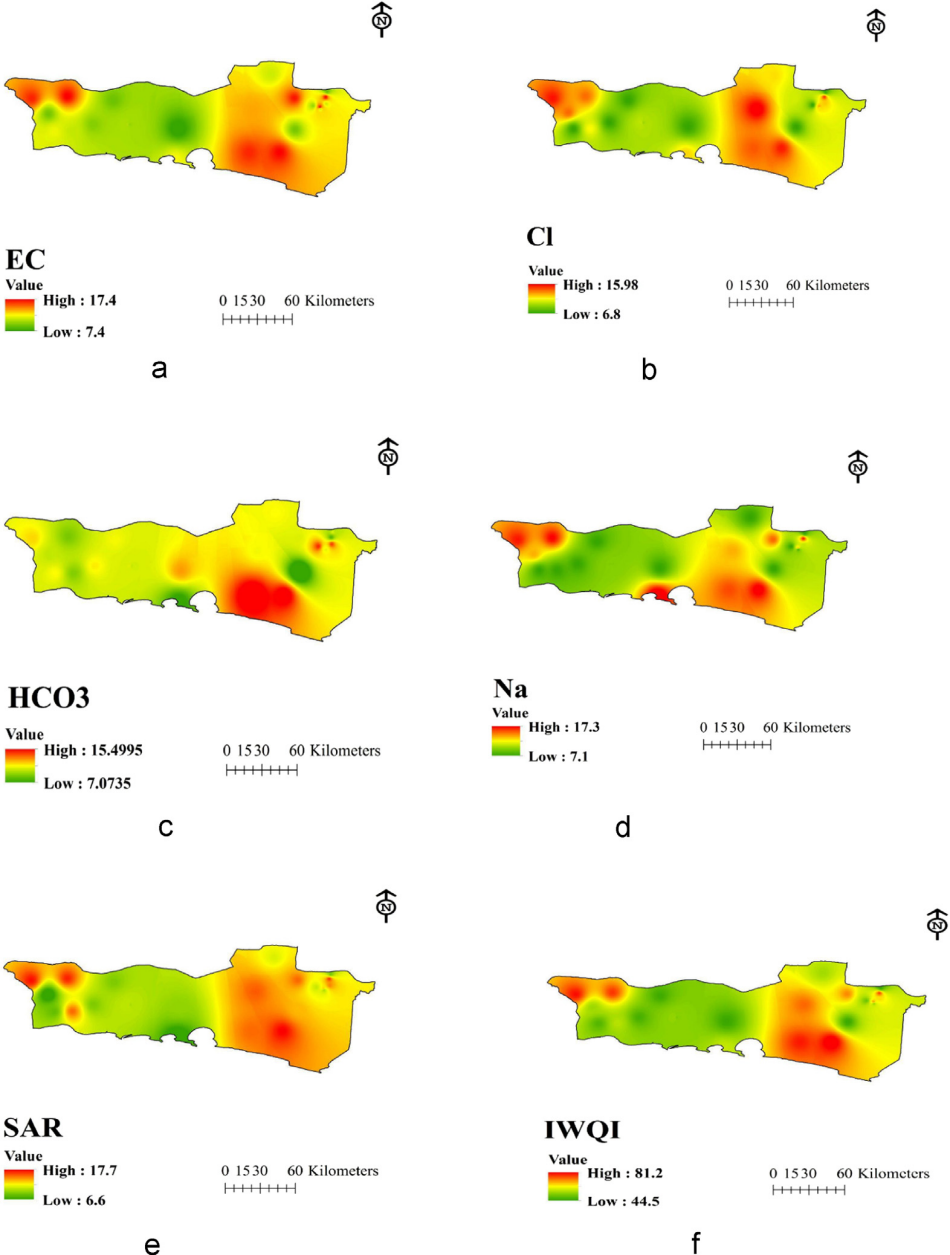
Spatial distribution maps (A) Q_i_*W_i_ of EC, (B) Q_i_*W_i_ of Cl^−^, (C) Q_i_*W_i_ of HCO_3_, (D) Q_i_*W_i_ of Na^+^, (E) Q_i_*W_i_ of SAR, (F) IWQI.

Chabahar city is located in Sistan and Baluchistan province of Iran encompassing an area of about 9739 km^2^ (Fig. 1) and its aquifers are located in South-East Iran between the latitudes 25°17´ N and longitudes 60°37´ E. The subjected study area is a semi-flat plain region with a gentle slope toward the south has a warm, temperate climate with an annual average of 25 °C in which the highest and lowest temperatures are 50 °C and −7 °C, respectively. This area was classified as a semiarid in which the precipitation ranges 70–130 mm per year with the evaporation rate of 4000 mm per year which is four times as high as Iran's average [6], [7] (Fig. 2 and Table 6).

**Table 6:** Water quality classification ranges and types of water based on IWQI values.

**Range**	**Type of groundwater**
<50	Excellent water
50–99.99	Good water
100–199.99	Poor water
200–299.99	Very poor water
≥300	Unsuitable for drinking/Irrigation purpose

### 2.2 Sample collection and analytical procedures

In this cross-sectional study, 40 rural drinking water sources in Chabahar villages in Sistan and Baluchistan province were selected and samples were collected during 12 months (2010–2011) and physical and chemical parameters were analyzed. The sampling locations of present study are illustrated in Fig. 1. Using polythene bottles (1 L) samples were collected and then transported to the central laboratory of the water and wastewater company. Groundwater samples were collected and transported to the laboratory on the same day and kept at 4 °C. All water samples were analyzed according to the Standard Methods for Examination of Water and Wastewater Temporary [1], [8], [9], [10]. Also permanent magnesium, calcium, and chloride were measured using titration method [1], [11], [12]. pH meter (model wtw, Esimetrwb) and turbidity meter (model Hach 50161/co 150 model P2100Hach, USA) are used to determine the concentration of hydrogen ion (pH) and electrical conductivity, respectively [1], [2], [3], [6], [10], [11], [12]. Finally, using GIS environment the numerical spatial distribution of the parameters were generated from analytical results and subsequently IDW (Inverse Distance weight) technique adopted to create the spatial distribution maps of water quality parameters and WQI. All analyses were done using Excel 2010 and Arc GIS 10.3 software.

### 2.3 Irrigation Water Quality Index (IWQIM)

IWQIM which is a specified method was developed primarily by Meireles et al. [5] and initially used for water quality assessment for agricultural purposes [5]. There are gentle differences between these methods and WQI based method was employed by WHO. In order to calculating relative weight in this method, estimated values of each parameter should be used which originating from the irrigation water quality data according to University of California Committee of Consultants (UCCC) as well as Ayers and Westcot Criteria [13]. However, In the IWQI model, firstly, the dominant parameters which play an important role in the water quality for agricultural purposes must be identified which are including EC, Na^+^, Cl^−^, and ${HCO}_{3}^{-}$ and SAR. In the second step, the weight of water quality parameters including: the water quality measurement parameter value (*Q_i_*), and the accumulation witness (*W_i_*) should be determined depending on each individual parameter value and finally taking account into the criteria which were proposed by Ayers and Westcot (Table 2) [13]. It has to be mentioned that in this model, lower value representing the poor quality of water and vice versa. Using the following equation, the value of Qi was calculated:

(1) $$q_{i}=q_{\max}-\left(\frac{[(x_{ij}-x_{\inf})\times q_{imap}}{x_{amp}}\right)$$

here, *q_max_* is the maximum value of *q*_i_ for each class; the observed value of each parameter is represented by *X_ij_*; also *X_inf_* refers the lower limit value of the class to which the parameter belongs; *q_imap_* represents the class amplitude and *X_amp_* is corresponds to class amplitude to which the parameter belongs. In this regard, the upper limit was considered to be the highest value determined in analysis of the water samples which is required in order to evaluate *X_amp_* of the last class of each parameter. Ultimately, *W_i_* values were normalized and their final sums equal one, according to Eq. (2):

(2) $$w_{i}=\frac{\sum_{j=1}^{k}F_{j}A_{ij}}{\sum_{j=1}^{k}\sum_{i=1}^{n}F_{i}A_{ij}}$$

The University of California Committee of Consultants (UCCC) estimated the values of (*q_i_*) according to factor amount, tolerance limit and irrigation water quality parameters which are summarized in Table 3. The parameters of water quality were the non-dimensional number and the higher of parameter value indicates the better of quality water. The results of water quality were determined at the laboratory.

Based on this equation, *w_i_* and *F* are corresponding to relative weight of the parameter for WQI and a constant value of component 1, respectively. *A_ij_* defines to what extent parameter *i* can be explained with factor *j*; *i* represents the number of physio-chemical and chemical parameters selected in IWQIM varied from 1 to *n* and *j* is the number of factors choose in IWQIM, ranged from 1 to *k*. Table 4 shows relative weight of each parameter. As a result of the above procedure the IWQIM value which is obtained from Eq. (3) and Table 5 indicated characteristics of IWQIM for each class.

(3) $$IWQIM=\overset{n}{\underset{i=1}{\sum }}q_{i}w_{i}$$

In this equation, IWQI is none dimensional Irrigation water quality index ranged from 0 to 100; *Q_i_* represents the quality of the parameter from 0 to 100 and corresponding to function of its measurement or concentration; *W_i_* refers the normalized weight of the parameter and related to the function of importance in explaining the global variability in water quality which are shown in Table 4. Based on existing water quality indexes, division in different classes based on the proposed water quality index has been carried out and considering the risk of salinity problems, soil water infiltration reduction, as well as toxicity to plants, classes were defined as observed in the classification presented by Bernardo [14] and Holanda and Amorim [4], [15]. Restriction to water use classes were characterized based on Meireles et al. (Table 5) [5].
